# Influenza and RSV make a modest contribution to invasive pneumococcal disease incidence in the UK

**DOI:** 10.1016/j.jinf.2013.02.007

**Published:** 2013-06

**Authors:** Emily J. Nicoli, Caroline L. Trotter, Katherine M.E. Turner, Caroline Colijn, Pauline Waight, Elizabeth Miller

**Affiliations:** a School of Social and Community Medicine, University of Bristol, Canynge Hall, 39 Whatley Road, Bristol BS8 2PS, UK; b Department of Mathematics, Imperial College London, South Kensington Campus, London SW7 2AZ, UK; c Immunisation Department, Health Protection Agency, Colindale Avenue, London NW9 5EQ, UK

**Keywords:** Invasive pneumococcal disease, Influenza, Respiratory syncytial virus, Seasonality

## Abstract

**Objectives:**

The common seasonality of incidence of invasive pneumococcal disease (IPD) and viral respiratory infections has long been recognized, however, the extent to which this affects the association between the pathogens is unknown. We have analysed weekly surveillance data of IPD, influenza and respiratory syncytial virus (RSV), using ambient temperature and hours of sunshine as measures of seasonality.

**Methods:**

Reported cases of influenza, IPD and RSV, were collected in England and Wales, from week 1 (January) 1996 to week 23 (June) 2009. The associations between IPD and respiratory viral infections were analysed using several statistical methods, including correlation coefficients and both additive and multiplicative regression models.

**Results:**

6–7.5% of cases of IPD are attributable to influenza and 3–4% attributable to RSV. Correlation coefficients reported considerably stronger associations between IPD and the viral infections compared to regression models.

**Conclusions:**

A small but potentially important percentage of IPD may be attributable to influenza and RSV when adjusted for seasonality by temperature. Jointly these viral infections may lead to over 10% of IPD cases. Therefore, prevention of viral respiratory infections may offer some additional benefit in reducing invasive pneumococcal infections.

## Introduction

The synergism between viral and bacterial infections has been widely reported, particularly for respiratory viruses and secondary bacterial pneumonia^1^; however, the underlying mechanisms are complex and remain largely unknown.^2,3^ Respiratory infections, such as influenza, respiratory syncytical virus (RSV) and *Streptococcus pneumoniae*, show strong seasonal patterns, each having increased incidence in winter in temperate areas of the world. Temperature, humidity, pollution, light intensity and increased crowding in winter^4–7^ have all been suggested as factors in causing the annual fluctuations in disease incidence. Despite many studies and the use of multiple statistical techniques, the strength of association between invasive pneumococcal disease (IPD) and respiratory viral infections remains unclear.

There has been a recent resurgence in interest in the relationship between IPD and influenza in the context of contemporary pandemic influenza preparedness and the use of the pneumococcal vaccines as an additional measure to prevent mortality.^8,9^ At a population level, several studies of surveillance data, outside of influenza pandemics, have sought to measure the associations between influenza, RSV and IPD.^4,5,10–19^ The reported strength of these associations varies between the studies, and appears to depend, at least partially, on the quantity of data available as well as the methods used. Even within the same data sample, the use of different statistical methods can lead to wildly different results.^10^ The associations are particularly difficult to measure because the common seasonality of the pathogens causes an overestimation of the result. A review of studies that have reported associations between IPD and influenza or RSV and their results can be found in the Supplementary Material.

We have conducted a novel analysis of IPD, influenza and RSV surveillance data from England and Wales, using a range of statistical methods, in order to estimate the proportion of IPD cases that are attributable to respiratory viruses, whilst attempting to account for the common seasonality of the pathogens.

## Methods

### Source of data

Clinically significant isolates of influenza,^20^ invasive pneumococcal disease (IPD)^21^ and respiratory syncytial virus (RSV) are recorded by microbiology laboratories in England and Wales. These are reported on a weekly basis to the Health Protection Agency (HPA) as part of the national surveillance system. We used data extracted from the HPA national surveillance database^22^ for influenza and RSV, and for IPD used a reconciled dataset as previously described.^21^ In brief, microbiology laboratories in England and Wales report all clinically significant pneumococcal isolates to the HPA through a computerized system (CoSurv). These isolates are often referred to the Respiratory and Vaccine Preventable Bacteria Reference Unit, HPA Microbiology Services for serotyping. These two datasets are then combined and any duplicates are removed.

Weekly counts of cases, between 1st January 1996 to 7th June 2009, stratified by age group (0–4 years, 5–14 years, 15–64 years and 65 years and over) were analysed. Average weekly temperatures (in degrees Celsius) and monthly sunshine (in hours) for the UK over the same period were sourced from the UK MET office information.^23^

### Statistical analyses

The relationship between the weekly incidence of IPD and viral infections was initially analysed by calculating the Pearson and Spearman's correlation coefficients, for the original and standardized datasets. The data were standardized in order to crudely remove the effect of the concurrent seasonality of the pathogens. For each weekly count, the data were standardized by subtracting the mean and dividing by the standard deviation of the counts for that week over all of the years of the study period (13 years), thus providing a measure of how the incidence for a particular week deviates from the average for that time of year.

Three different regression models were investigated (Table 2). Two were additive models (a basic linear regression and an identity-linked negative binomial regression) and one was a multiplicative model (a log-linked negative binomial regression). The negative binomial regression models were applied to account for over-dispersion of the dataset. The dependent variable was the incidence of IPD, with explanatory variables, the incidence of influenza and of RSV. Two additional explanatory variables, the UK mean weekly temperature^23^ and monthly hours of sunshine, were investigated in the models to adjust for the common seasonality of the pathogens. The models were applied to all ages and then to each age group individually, as well as to a range of lags (0–4 weeks).

We estimated the percentage of IPD cases that could be attributable to influenza and RSV. For the additive models, this was estimated by multiplying the virus' case count with its regression coefficient. This determined the estimated number of cases of IPD attributable to the virus and from which a percentage could be calculated. For the multiplicative model, the percentages of IPD cases attributable to influenza and RSV were estimated by multiplying the virus' case count with its fitted rate ratio (RR), (attributable percentage = case count × (RR-1) × 100).^24^

All analyses were carried out with STATA version 11.2 (StataCorp. 2009. *Stata Statistical Software: Release 11*. College Station, TX: StataCorp LP).

## Results

**Figure 1:**
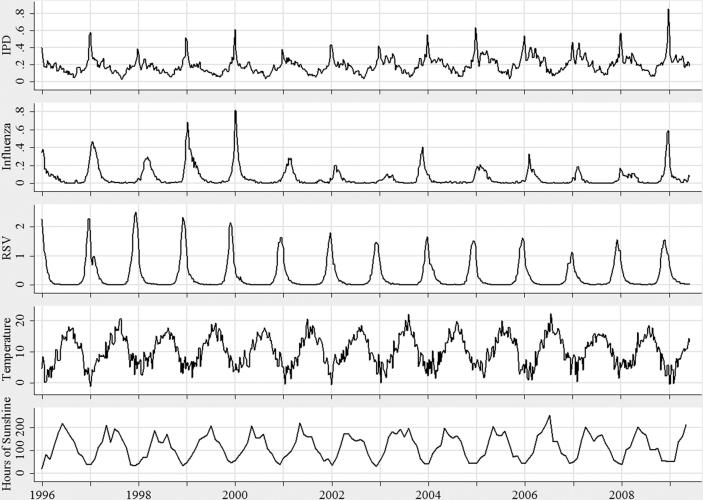
Time series plots of incidence per 100 000 population of IPD, influenza and RSV, the mean weekly temperature (°C) and monthly sunshine (h), from week 1 (January), 1996 to week 23 (June), 2009.

**Figure 2:**
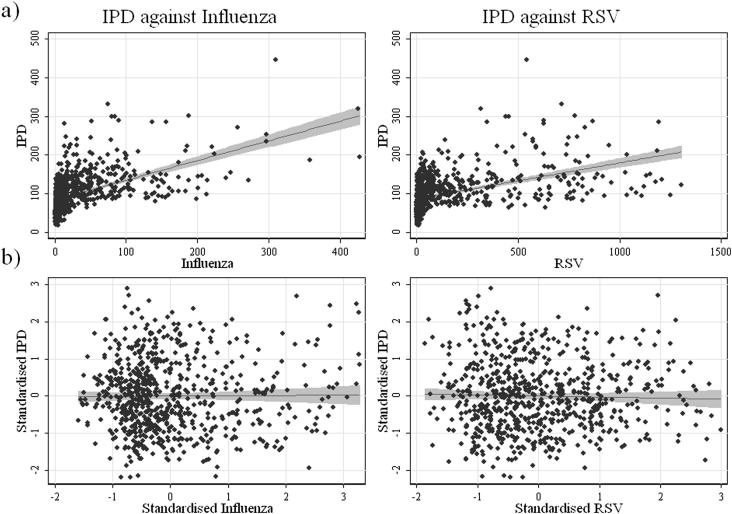
Scatter plot of (a) weekly incidence of IPD against weekly incidence of viral infection (influenza [LEFT] and RSV [RIGHT]) and (b) the equivalent standardized results; with linear trend lines and 95% confidence intervals.

The common seasonal incidence of all three diseases, IPD, influenza and RSV can be clearly seen in this dataset (Fig. 1). Whilst IPD cases are reported all year round, there are distinct increases during winter months. For influenza, there are similarly timed peaks in reported incidence, but with fewer cases out of season. The same is true for RSV, with very few cases reported in the summer months and with large numbers of cases being reported, mainly in infants (Table 1), in the winter. Fig. 2a also displays the strong shared seasonality of the different pathogens. However, in Fig. 2b, where the data have been standardized to remove the effect of the common seasonality, the correlation in the scatter plot is weakened considerably compared to Fig. 2a. The age group with the largest reported incidence of IPD is the over 65 year olds (49.0%). The 0–4 year age group reported the most RSV infections (94.8%). For influenza, most cases were reported in the 15–64 years age group (49.6%).

**Figure 3:**
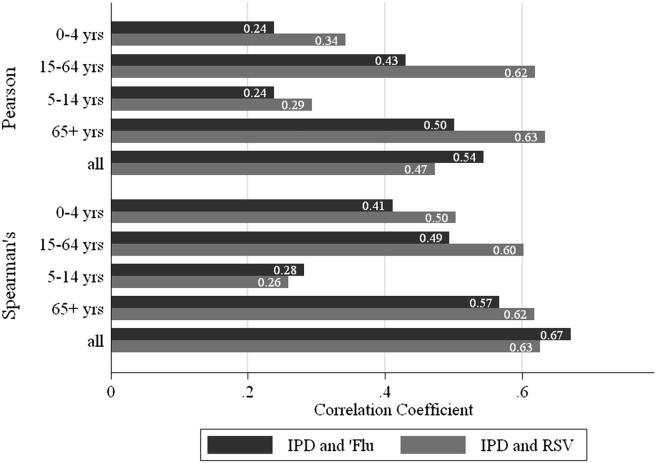
Pearson and Spearman's correlation coefficients for all ages and each age group for IPD and influenza and IPD and RSV (all coefficients have *P*-values <0.001).

Pearson and Spearman's correlation coefficients between IPD and both respiratory viruses found strong, significant associations for all age groups (Fig. 3): all coefficients have a *P*-value <0.001. In most age groups, the correlation coefficients are higher for RSV than for influenza. Both coefficients are highest in the older age groups, with the 65 years and over having the strongest correlation for IPD and influenza and similarly strong associations for IPD and RSV.

In the multivariate regression analyses, the factor responsible for the strongest associations with IPD is found to be the average temperature as opposed to either of the viral infections or hours of sunshine (Tables 3 and 4). There was no evidence of an association between IPD and hours of sunshine (results not shown). There was, however, some evidence of an association between IPD and one month lagged hours of sunshine (Table 4). For the age group of all ages, the strongest viral association is with influenza, followed by RSV, for all of the regression techniques. There is no evidence of any significant time lags in the incidence data (i.e. model fit did not improve with the introduction of any lags of 1–4 weeks).

The linear regression model adjusted by weekly temperature indicates that 6.9% of IPD cases are attributable to influenza and 3.9% attributable to RSV, for all ages (Table 5). The results using the additive negative binomial model are similar (7.5% attributable to influenza and 3.5% attributable to RSV) and the results from the multiplicative negative binomial model are slightly lower than the additive models (5.6% attributable to influenza and 2.9% attributable to RSV). For the linear model adjusted by lagged monthly sunshine, 6.1% of IPD cases were attributable to influenza and 3.8% attributable to RSV, for all ages (Table 6). The percentage is higher for the additive negative binomial model (9.2% attributable to influenza and 4.1% attributable to RSV) and lower for the multiplicative negative binomial model (5.7% attributable to influenza and 3.4% attributable to RSV). The multiplicative model tends to predict a lower percentage of attributable IPD cases to influenza and RSV in all of the age groups.

For RSV, the lowest percentage of attributable cases is in the 0–4 year olds (1–2%, dependent on the model) and the highest percentage is in the 15–64 year olds (15–25%). The percentages of attributable IPD cases increase across all age groups and in all models. The percentage of influenza-attributable cases increased with age from 0 to 6%. The percentage of cases attributable to RSV ranged from 3 to 28% with the highest percentage being for the 15–64 years group.

## Discussion

We have found a small but statistically significant association between invasive pneumococcal disease and viral infections after accounting for the common seasonality of the infections. Influenza-attributable IPD accounted for between 0 and 9.2% of cases of IPD according to age, meteorological variable and regression method used. In the additive negative binomial regression model, 7.5% of IPD is attributable to influenza, for all ages, when adjusted by average temperature (best-fitting model). The percentage of RSV-associated IPD accounted for between 1.5 and 25% of all IPD cases, with 3.5% of IPD attributable to RSV, for all ages, when adjusted by average temperature in the additive negative binomial regression model. Our results for influenza are in line with those of other studies applying similar techniques. They found influenza was associated with 6–10%^11^ and 5–6%^17^ of IPD cases. Our study is the first, to our knowledge, to estimate the IPD cases attributable to both influenza and RSV, in different age groups and including average temperature and hours of sunshine to allow for the seasonal characteristics of the data.

Our study has looked in more detail at the influence of age in associations between IPD and viral infections. We found that for influenza the attributable percentage of IPD cases is lowest in the 0–4 years group for both meteorological variables (∼0%) and highest in the over 65 years group when adjusted by temperature (3.2–4.8%, dependent on the model) or highest in the 5–14 years group when adjusted by hours of sunshine (5.7–6.9%). For RSV, the attributable percentage of IPD cases was again lowest in the 0–4 years group for both meteorological variables (1–2%) and highest in the 15–64 years group for both variables (14.5–25%). In previous studies, evidence of associations between influenza and IPD has been more consistently reported in adults^11,13,14,17^ compared to children where the associations are weaker or non-existent.^4,5,12,15,16^ We also found that the associations between IPD and influenza were stronger in older age groups when adjusted by temperature. This was not the case when adjusted by hours of sunshine. However the data on hours of sunshine is only available at monthly time periods as opposed to weekly temperature measurements and the association between IPD and temperature was found to be stronger than that between IPD and sunshine (where all data was converted to monthly time periods).

In the case of IPD and RSV in children, most studies^4,5,18^ have found the association between IPD and RSV was stronger than that of IPD and influenza, with only Talbot et al.^15^ finding the reverse result. Our study also estimates that more cases of IPD in children are attributable to RSV than influenza; however the strength of the statistical evidence of our results for influenza is weak. We also found a similar result for adults.

Whether the average air temperature and hours of sunshine are merely markers of some other underlying process or whether they do truly affect the transmissibility, severity or duration of infection is still debated. However, due to the relative strength of the evidence that average temperature^18,25–30^ and hours of sunshine,^6,14,18,31–35^ are associated with IPD and viral infections it was determined that they were a logical choice to account for seasonality. We found a 1 month lag in the association between IPD and hours of sunshine, consistent with three other studies reporting lags of 2–5 weeks^6,14,33^ though no lag was reported in 2 other studies.^31,32^ This may be related to the strong, positive effects of sunlight on the immune system due to increased 1,25-(OH)_2_-vitamin-D metabolism.^35,36^ Other meteorological factors such as rainfall and relative humidity were not included in the models as associations with IPD and viral infections are less consistent.^14,18,27,31^ It may be that the use of average temperature as an adjustment for seasonality has led to slightly lower percentages, for some age-groups, of influenza-attributable IPD when compared to previous studies which included seasonal harmonic curves.^11,17^ However, the use of harmonic curves does not allow for annual variations.

From our results using Pearson and Spearman's correlation coefficients, we could conclude that there is a very strong association between IPD and the viral infections; however these are rather crude measures of association that cannot be seasonally adjusted, and so are likely to overestimate any association in our data. Further analysis, beyond the use of correlation coefficients, should be considered in similar studies of seasonal diseases in order to formulate more robust conclusions. We investigated a range of regression models; looking at both additive and multiplicative models. It is considered that the additive model is a more plausible fit for this biological data,^37^ a multiplicative relationship between the independent variable terms in the model would be hard to substantiate. However, it is difficult to firmly conclude which model is the best as we have no gold standard for comparison (see ref and below).^10^

The ecological nature of this study restricts the conclusions that can be drawn. Research at an individual level may be more revealing with respect to the true incidence of virus-attributable IPD, but will be more challenging. Potential study designs that could be employed include case-control studies of IPD with serological investigations of recent viral infections. There are further limitations in the use of surveillance systems for the data in this study, under-reporting and changes over time in the reporting thresholds cannot be ruled out. The lack of distinction between the strains and serotypes of the diseases could mask any true associations between IPD and the viral infections, if the association only applies to a subgroup of the strains or serotypes.^38^ There are important issues with the statistical methods used to gauge the associations, other studies^10,38^ have found, as we have, that the different methods produce variable results. Moreover, negative binomial regression requires assumptions to be made about the data; the observations should be independent and the virulence of the viruses should remain constant.

The possible mechanisms underlying the interaction between *S. pneumoniae* and influenza and RSV have been reviewed by Bosch et al.^39^ A primary host defence to infection is the secretion of a mucus layer in the upper respiratory tract. Bacteria bind to the mucus^40,41^ enabling them to be cleared by the action of cilia cells. However, primary viral infection destroys these epithelial cells through metabolic exhaustion or lysis^39^ reducing mucus and bacterial clearance.^42^ This enables bacteria to progress further into the respiratory tract by inhalation or adherence to exposed cell surface receptors.^43,44^ Viral factors produced by influenza and RSV also increase bacterial adherence. Influenza produces neuraminidase (NA), which cleaves sialic acids exposing bacterial receptors and thus increasing adherence.^45^ RSV expresses RSV-protein G which acts directly as a bacterial receptor.^46^ Viral infection may alter behaviour of the immune system, by modifying the expression of antimicrobial peptides^47^ and adhesion proteins, these act as receptors for immune cells, however *S. pneumoniae* and other bacteria have been shown to adhere to these proteins as well.^48,49^ Influenza virus is also known to impair neutrophil function and increase apoptosis,^50^ decrease oxidative burst^51^ and reduce production and activity of cytokines.^39^

The time period of our analysis covers only seasonal influenza and excludes the H1N1 ‘swine flu’ pandemic. We censored our dataset at the week preceding the World Health Organization's (WHO) declaration of the pandemic on 11th June 2009 because the UK surveillance systems were modified and enhanced during the pandemic, making direct comparisons with previous time periods difficult. During the second wave of the pandemic in winter 2010/2011, linkage between influenza and invasive bacterial infection surveillance reports suggested that between 6 and 11% (depending on age, with the highest percentage in the 15–44 year age group) of IPD cases had concurrent influenza.^52^ This is broadly consistent with our findings.

We suggest that there is a small, but measurable association between IPD and RSV and influenza. These results are relevant for public health policy decision making. Prevention of viral respiratory infections may offer some additional benefit in terms of reducing invasive pneumococcal infections^53^ and prevention of pneumococcal infections during, say, influenza pandemics could see a reduction in hospitalizations and mortality.^8,9^ There would be merit in considering both IPD and viral infections when looking at interventions for any one of these infections and this could be explored using modelling techniques.^54^

## Figures and Tables

**Table 1:** Number (percentage), by age group, of cases of each disease over the study period.

Age group	IPD	Influenza	RSV
0–4 yrs	8153 (11.43%)	4510 (18.30%)	107 525 (94.81%)
5–14 yrs	1787 (2.51%)	2393 (9.71%)	1190 (1.05%)
15–64 yrs	26 457 (37.09%)	12 209 (49.55%)	3079 (2.72%)
65+ yrs	34 936 (48.98%)	5528 (22.44%)	1616 (1.43%)
*Total*	*71 333*	*24 640*	*113 410*

**Table 2:** Description of regression models, method, log- and identity-linked and their equation.

Model	Method	Link	Equation
1	Linear	Identity	*Y* = *β*_0_ + *β*_1_·*X*_FLU_ + *β*_2_·*X*_RSV_ + *β*_3_·*X*_TEMP_
2	Negative binomial	Identity	*Y* = *β*_0_ + *β*_1_·*X*_FLU_ + *β*_2_·*X*_RSV_ + *β*_3_·*X*_TEMP_
3	Negative binomial	Log	log(*Y*) = *β*_0_ + *β*_1_·*X*_FLU_ + *β*_2_·*X*_RSV_ + *β*_3_·*X*_TEMP_

**Table 3:** Linear regression, negative binomial regression with an identity link and with a log link for each age group with temperature as a meteorological variable.

Age	Variable	Additive				Multiplicative	
		Linear Reg.		N. Binomial Reg. with identity link		N. Binomial Reg. with log link	
		Coefficient	*P*-value	Coefficient	*P*-value	Coefficient	*P*-value
All ages	‘Flu	0.262	<0.001	0.285	<0.001	0.002	<0.001
	RSV	0.032	<0.001	0.029	<0.001	0.000	0.001
	Temp.	−4.711	<0.001	−4.664	<0.001	−0.060	<0.001
	Constant	136.102	<0.001	135.279	<0.001	5.095	<0.001

0–4 yrs	‘Flu	−0.004	0.771	0.004	0.787	−0.001	0.577
	RSV	0.002	0.046	0.002	0.032	0.000	0.135
	Temp.	−0.613	<0.001	−0.630	<0.001	−0.060	<0.001
	Constant	17.746	<0.001	17.848	<0.001	3.024	<0.001

5–14 yrs	‘Flu	0.033	0.023	0.032	0.076	0.009	0.090
	RSV	0.132	<0.001	0.109	0.001	0.034	0.001
	Temp.	−0.084	<0.001	−0.094	<0.001	−0.043	<0.001
	Constant	3.081	<0.001	3.227	<0.001	1.246	<0.001

15–64 yrs	‘Flu	0.092	0.002	0.071	0.064	0.001	0.098
	RSV	1.853	<0.001	1.640	<0.001	0.032	<0.001
	Temp.	−1.158	<0.001	−1.401	<0.001	−0.048	<0.001
	Constant	40.006	<0.001	43.771	<0.001	3.896	<0.001

65+ yrs	‘Flu	0.340	<0.001	0.406	<0.001	0.004	<0.001
	RSV	2.647	<0.001	2.502	<0.001	0.034	<0.001
	Temp.	−2.407	<0.001	−2.298	<0.001	−0.062	<0.001
	Constant	65.960	<0.001	64.553	<0.001	4.366	<0.001

**Table 4:** Linear regression, negative binomial regression with an identity link and with a log link for each age group with monthly lagged hours of sunshine as a meteorological variable.

Age	Variable	Additive				Multiplicative	
		Linear Reg.		N. Binomial Reg. with identity link		N. Binomial Reg. with log link	
		Coefficient	*P*-value	Coefficient	*P*-value	Coefficient	*P*-value
All ages	‘Flu	0.226	<0.001	0.349	<0.001	0.000	<0.001
	RSV	0.030	0.006	0.033	0.005	0.000	0.013
	Sun	−1.655	<0.001	−1.320	<0.001	−0.004	<0.001
	Constant	577.816	<0.001	519.261	<0.001	6.456	<0.001

0–4 yrs	‘Flu	−0.004	0.879	0.013	0.571	0.000	0.832
	RSV	0.002	0.293	0.002	0.255	0.000	0.415
	Sun	−0.213	<0.001	−0.213	<0.001	−0.005	<0.001
	Constant	74.342	<0.001	73.834	<0.001	4.427	<0.001

5–14 yrs	‘Flu	0.060	0.014	0.066	0.029	0.004	0.017
	RSV	0.149	0.008	0.147	0.005	0.010	0.010
	Sun	−0.015	0.235	−0.018	0.084	−0.002	0.036
	Constant	10.881	<0.001	11.165	<0.001	2.500	<0.001

15–64 yrs	‘Flu	0.043	0.414	0.094	0.157	0.000	0.407
	RSV	2.396	<0.001	2.752	<0.001	0.011	<0.001
	Sun	−0.237	0.050	−0.168	0.059	−0.003	<0.001
	Constant	142.349	<0.001	125.034	<0.001	5.105	<0.001

65+ yrs	‘Flu	0.204	0.010	0.447	0.006	0.001	0.079
	RSV	3.800	<0.001	4.509	<0.001	0.013	<0.001
	Sun	−0.692	<0.001	−0.503	<0.001	−0.004	<0.001
	Constant	251.428	<0.001	215.461	<0.001	5.633	<0.001

**Table 5:** Percentage of IPD cases attributable to influenza and RSV for the two additive models; linear regression model and negative binomial regression model with identity-link (additive) and with log-link (multiplicative) with temperature as a meteorological variable.

Age	Attributable to	Linear regression				Additive negative binomial regression				Multiplicative negative binomial regression			
		%	Std dev^a^	95% CI^b^		%	Std dev^a^	95% CI^b^		%	Std dev^a^	95% CI^b^	
All ages	Influenza	6.888	8.114	0.461	26.312	7.461	8.689	0.503	28.131	5.557	8.745	0.158	23.806
	RSV	3.892	5.734	0.167	17.731	3.525	5.235	0.150	16.175	2.940	4.779	0.054	14.208

0–4 yrs	Influenza	−0.198	0.410	−0.932	0.000	0.184	0.374	0.000	0.868	−0.373	0.785	−1.793	0.000
	RSV	1.764	2.766	0.038	8.338	1.956	3.052	0.044	9.175	1.418	2.364	0.018	6.949

5–14 yrs	Influenza	3.493	5.166	0.000	14.782	3.359	4.975	0.000	14.171	2.919	5.103	0.000	13.644
	RSV	6.598	9.160	0.000	26.898	5.548	7.815	0.000	22.849	5.867	9.415	0.000	27.568

15–64 yrs	Influenza	3.441	4.005	0.197	12.552	2.667	3.150	0.151	9.941	1.831	2.770	0.105	7.441
	RSV	16.712	13.898	0.000	44.124	14.911	12.609	0.000	40.078	14.514	17.138	0.000	52.721

65+ yrs	Influenza	4.047	5.593	0.000	16.328	4.781	6.502	0.000	19.176	3.226	6.306	0.000	14.687
	RSV	9.514	9.365	0.000	28.468	9.053	8.962	0.000	27.222	7.905	10.236	0.000	27.356

a Standard deviation.

b 95% confidence interval.

**Table 6:** Percentage of IPD cases attributable to influenza and RSV for the two additive models; linear regression model and negative binomial regression model with identity-link (additive) and with log-link (multiplicative) with monthly lagged hours of sunshine as a meteorological variable.

Age	Attributable to	Linear regression				Additive negative binomial regression				Multiplicative negative binomial regression			
		%	Std dev^a^	95% CI^b^		%	Std dev^a^	95% CI^b^		%	Std dev^a^	95% CI^b^	
All ages	Influenza	6.127	6.925	0.723	19.907	9.233	9.907	1.172	29.012	5.740	8.462	0.414	23.312
	RSV	3.751	5.284	0.193	16.457	4.122	5.824	0.213	17.789	3.393	5.220	0.096	15.328

0–4 yrs	Influenza	−0.184	0.348	−0.717	0.000	0.562	1.030	0.000	2.210	−0.221	0.423	−0.917	0.000
	RSV	1.580	2.368	0.052	7.066	1.612	2.409	0.055	7.215	1.213	1.913	0.024	5.638

5–14 yrs	Influenza	6.377	8.142	0.000	23.730	6.899	8.673	0.000	25.393	5.686	8.849	0.000	23.850
	RSV	7.944	9.531	0.000	29.074	7.724	9.267	0.000	28.360	7.600	10.624	0.000	34.290

15–64 yrs	Influenza	1.655	1.881	0.218	5.888	3.581	3.896	0.493	12.417	1.408	1.990	0.112	4.918
	RSV	22.212	15.883	2.228	53.665	25.085	17.146	2.658	58.155	20.826	22.660	1.091	73.080

65+ yrs	Influenza	2.533	3.295	0.242	9.416	5.322	6.429	0.545	19.129	2.213	3.985	0.129	8.642
	RSV	14.120	10.792	0.000	35.426	16.574	12.330	0.000	40.386	12.977	14.289	0.000	42.692

a Standard deviation.

b 95% confidence interval.
