# Supplementary material for: Influenza and RSV make a modest contribution to invasive pneumococcal disease incidence in the UK
Source: J Infect. 2013 Jun;66(6):512–20. doi: 10.1016/j.jinf.2013.02.007 (PMC3650581; doi:10.1016/j.jinf.2013.02.007)
Supplement: Supplementary file 1 [file mmc1.docx]

SUPPLEMENTARY MATERIAL

**Supplementary Table 1** - Review of studies relating to ecological retrospective studies looking at associations between IPD and influenza or IPD and RSV

| **Author, Date** | **Study Year** | **Population** | **Data Sources** | **Statistical Technique** | **Summary of Findings** |
| --- | --- | --- | --- | --- | --- |
| Ampofo et al. 2008 ([4](#_ENREF_4)) | 2001-2007 | Children <18 in Salt Lake City, Utah, USA | Culture-confirmed and ICD-9 coded IPD and lab-confirmed viruses at Children’s Medical Centre | Pearson correlation coefficient | Strong association between IPD and RSV and between IPD and influenza for both Culture-confirmed and ICD-9 coded IPD |
| Gilca et al. 2009 ([10](#_ENREF_10)) | 2001-2003 | Infants <3 in Quebec, Canada | Hospital nasopharyngeal aspirates | Serfling regression, periseason differences, generalized linear models, Box-Jenkins model | The accuracy of these statistical methods is unknown. |
| Grabowska et al. 2006 ([11](#_ENREF_11)) | 1994-2004 | Sweden  all ages | Swedish Institute for Infectious disease Control and weekly serology reports | Negative binomial regression | Association between IPD and influenza, with 3 week lag. 12-20% of IPD due to previous ‘flu |
| Jansen et al. 2008 ([12](#_ENREF_12)) | 1997-2003 | The Netherlands  all ages | Sentinel System for Clinical Virology for influenza & RSV. Reference Laboratory for Bacterial Meningitis for IPD | Spearman’s correlation coefficient corrected for long term trends & incidence rate ratios. | During periods of influenza or RSV predominance, rates of IPD were higher in older children and adults, but not in young children |
| Kim et al. 1996 ([5](#_ENREF_5)) | 1990-1993 | Houston, USA  all ages | Community surveillance system | Pearson correlation coefficient & multivariate regression | Clear temporal association between IPD & ‘flu and IPD & RSV. Correlations with ambient temperature |
| Kuster et al. 2011 ([19](#_ENREF_19)) | 1995-2009 | Toronto, Canada all ages | Toronto Invasive Bacterial Diseases Network and Public Health Agency of Canada | Fast fourier transforms, negative binomial regression, Granger-causality test and case-crossover methods | ‘Flu granger-causes IPD. Short term association between ‘flu and IPD with 1 week lag |
| Kyaw et al. 2002 ([13](#_ENREF_13)) | 1988-1999 | Scotland  all ages | Scottish Centre for Infection & Environmental Health & Scottish Pneumococcus lab | Figure comparing quarterly incidence | Cases of IPD and influenza peaked in the 1^st^ three months of the year |
| Murdoch et al. 2009 ([14](#_ENREF_14)) | 1995-2006 | Christchurch, New Zealand | Canterbury Health Laboratories information system | Spearman’s correlation coefficient and negative binomial regression | IPD & RSV association in children <5. IPD & ‘flu association in >65 |
| Talbot et al. 2005 ([15](#_ENREF_15)) | 1995-2002 | 5 Tennessee counties, USA  all ages | Weekly IPD from CDC ABCs & prospective lab-based surveillance for viruses | Pearson correlation coefficient & one-sample test of proportion | Correlation between IPD & RSV & IPD & influenza, greater in adults than children |
| Toschke et al. 2008 ([16](#_ENREF_16)) | 1997-2003 | Children <16 in Germany | Active surveillance data from pediatric hospitals. Monthly counts of isolated IPD and influenza A | Farrington outbreak identification and ‘3h algorithm’ | No temporal association between IPD and influenza A in children was found |
| Walter et al. 2010 ([17](#_ENREF_17)) | 1995-2006 | 6 – 9 states of the USA  all ages | Weekly IPD from CDC ABCs and WHO laboratories for influenza | Seasonally adjusted negative binomial regression | Modest association between IPD & influenza. 11-14% of IPD occurring in high ‘flu weeks, may have been associated with ‘flu |
| Watson et al. 2006 ([18](#_ENREF_18)) | 2000 | New South Wales, Australia,  all ages | New South Wales Pneumococcal Network & respiratory virus infections surveillance | Pearson correlation coefficient | No significant correlation between influenza & IPD. Correlation with IPD and RSV in children. Strong association with temperature, not with humidity, wind speed or rainfall. |
